# ID 309 - Cenário da Doença Renal Crônica na Perspectiva das Diretrizes Clínicas e da Avaliação de Tecnologias

**DOI:** 10.5327/2237-9622.2025.v34s1.309

**Published:** 2025-11-25

**Authors:** Cynthia Carolina Duarte Andrade, Amanda Oliveira Lyrio, Dalila Fernandes Gomes, Gláucia Teles de Araújo Bueno, Marta da Cunha Lobo Souto Maior

**Keywords:** insuficiência renal crônica, protocolos clínicos, diagnóstico, tecnologia, Sistema Único de Saúde

## Abstract

**Introdução:**

Há inovações tecnológicas constantes no cuidado do paciente com doença renal crônica (DRC). Nos últimos dez anos, cinco diretrizes clínicas sobre DRC foram aprovadas pela Comissão Nacional de Incorporação de Tecnologias no Sistema Único de Saúde (Conitec), as quais norteiam o diagnóstico, o tratamento e o monitoramento desses pacientes, considerando os medicamentos disponíveis no Sistema Único de Saúde (SUS). Este estudo tem por objetivo descrever a linha do tempo das atualizações e a utilização dos medicamentos incorporados.

**Método:**

Foram revisadas cinco diretrizes clínicas sobre a DRC, bem como os medicamentos incorporados. A partir dos registros das dispensações de medicamentos do Componente Especializado da Assistência Farmacêutica (Ceaf), realizadas de janeiro de 2014 a junho de 2024, extraídos da Sala Aberta de Situação de Inteligência em Saúde (Sabeis), foi obtido o número anual de pacientes por medicamentos. Foram incluídas dispensações associadas aos códigos da Classificação Estatística Internacional de Doenças e Problemas Relacionados com a Saúde (CID-10) de DRC N18.2, N18.0, N18.8, N18.3, N18.4 e N25.0. Foram excluídos os registros sem identificação criptografada dos pacientes ou sem quantidade aprovada.

**Resultados:**

Em 2014, foram publicadas as Diretrizes Clínicas para o Cuidado ao Paciente com DRC no SUS. Em 2015, os medicamentos paricalcitol e o cinacalcete foram incorporados para pacientes com hiperparatireoidismo secundário (HPTS) à DRC3. Em 2017, com a publicação do Protocolo Clínico e Diretrizes Terapêuticas (PCDT) do Distúrbio Mineral Ósseo na DRC2, duas diretrizes anteriores foram unificadas. Após cinco anos, esse PCDT foi atualizado, com a ampliação de uso desses medicamentos por pacientes com HPTS à DRC 5D4. Em 2023, a carboximaltose férrica foi incorporada para o tratamento de pacientes adultos com anemia por deficiência de ferro e intolerância ou contraindicação aos sais orais de ferro, e a Conitec aprovou a atualização do PCDT de Anemia na DRC, com a unificação dos existentes (alfaepoetina e de reposição de ferro). Em 2023, a Conitec também aprovou o PCDT das Estratégias para atenuar a progressão da DRC, o qual já considera a incorporação de dapagliflozina para tratamento de pacientes adultos com DRC em uso de terapia-padrão.

Esses esforços se refletiram no aumento de 28,03% (de 158.492 para 202.924) no número de pacientes que receberam medicamentos para tratar a DRC, totalizando 613.667 pacientes no período. Em 2024, a maioria dos pacientes utilizou alfaepoetina (41%), sevelâmer (23%) e sacarato de hidróxido férrico (15%). O uso de alfacalcidol e calcitriol apresentou queda acentuada, e o calcitriol foi utilizado por 7%. Após a publicação do PCDT do Distúrbio Mineral Ósseo na DRC2, o uso de cinacalcete e paricalcitol aumentou anualmente, representando 7% dos pacientes cada um em 2024. Não foram encontrados registros sobre o uso de dapagliflozina.

**Conclusão:**

As recomendações da Conitec conciliam a incorporação racional e o acesso à inovação tecnológica para os cidadãos brasileiros. A análise temporal permitiu observar que os PCDT orientam o acesso aos medicamentos incorporados, promovendo o cuidado integral ao paciente com DRC.

